# Mild volume acute normovolemic hemodilution is associated with lower intraoperative transfusion and postoperative pulmonary infection in patients undergoing cardiac surgery -- a retrospective, propensity matching study

**DOI:** 10.1186/s12871-017-0305-7

**Published:** 2017-01-26

**Authors:** Zhen-feng Zhou, Xiu-ping Jia, Kai Sun, Feng-jiang Zhang, Li-na Yu, Tian Xing, Min Yan

**Affiliations:** 1 Department of Anesthesiology, Second Affiliated Hospital, School of Medicine, Zhejiang University, Zhejiang Province, China; 2 Department of Anesthesiology, The Affiliated Yiwu Hospital of Wenzhou Medical University, Yiwu, China

**Keywords:** Hemodilution, Transfusion, Complication, Cardiac surgery

## Abstract

**Background:**

Perioperative allogenic transfusion is required in almost 50% of patients undergoing cardiac surgery and is associated with higher risk of mortality and morbidity (Xue et al., Lancet 387:1905, 2016; Ferraris et al., Ann Thorac Surg 91:944–82, 2011). Acute normovolemic hemodilution (ANH) is recommended as a potential strategy during cardiac surgery, but the blood conservation effect and the degree of ANH was still controversial. There is also an increasing concern about the improved outcomes associated with ANH. Therefore, a better understanding of the effect of mild volume ANH during cardiac surgery is urgently needed.

**Methods:**

This retrospective study included 2058 patients who underwent cardiac surgery between 2010 and 2015. The study population was split into two groups (with and without mild volume ANH). Propensity score adjustment analysis was applied. We reported the association between the use of mild volume ANH and perioperative outcomes.

**Results:**

A total of 1289 patients were identified. ANH was performed in 358 patients, and the remaining 931 patients did not receive any ANH. Five hundred of the total patients (38.8%) received perioperative RBC transfusions, 10% (129/1289) of patients received platelet, and 56.4% (727/1289) of patients received fresh frozen plasma transfusions. Mild volume ANH administration was significantly associated with decreased intraoperative RBC transfuse rate (8.5% vs. 14.4%; *p* = 0.013), number of RBC units (*p* = 0.019), and decreased postoperative pulmonary infection (6.8 vs. 11.3%; *p* = 0.036) during cardiac surgery. However, there was no significant difference regarding intraoperative fresh frozen plasma (FFP) and platelet concentrate transfusions, as well as postoperative and total perioperative allogeneic transfusions. Furthermore, there was no significant difference regarding postoperative outcomes including mortality, prolonged wound healing, stroke, atrial fibrillation, reoperation for postoperative bleeding and acute kidney injury. There was also no difference in postoperative ventilation time, length of ICU and hospital stay.

**Conclusion:**

Based on the 5-year experience of mild volume ANH in cardiac surgeries with CPB in our large retrospective cohort, mild volume ANH was associated with decreased intraoperative RBC transfusion and postoperative pulmonary infection in Chinese patients undergoing cardiac surgery. However, there was no significant difference regarding postoperative and total perioperative allogeneic transfusions.

**Electronic supplementary material:**

The online version of this article (doi:10.1186/s12871-017-0305-7) contains supplementary material, which is available to authorized users.

## Background

In China, blood shortage is now becoming an important public health concern, and the lack of adequate blood donation is considered to be the main cause [1]. Perioperative allogenic transfusion is required in about 50% of patients undergoing cardiac surgery [2], but it is associated with higher risk of mortality and morbidity (infection, lung injury, renal failure, and stroke) [3–5]. Hence, many blood conservation techniques are recommended of which acute normovolemic hemodilution (ANH) may be considered as an adjuvant in appropriate patients [2].

Thus far, no consensus has been achieved in the results of published studies on ANH. Many trials have confirmed the positive effects of ANH in reducing perioperative allogeneic transfusions [6, 7]; however, other studies have reported negative effects [8, 9]. Another important debated point is the degree of ANH performed. Some authors consider that only a large volume of ANH is useful or effective in minimizing the need for allogeneic transfusions [7, 10]; however, Kahraman et al. observed no differences between the effect of mild and high volume of ANH on postoperative bleeding and perioperative blood transfusions [11]. Furthermore, there may be a risk of performing moderate or severe ANH in patients with a lower preoperative hematocrit level in cardiac surgery with cardiopulmonary bypass (CPB) which would lead to hemodilution [12].

There is also an increasing concern about the improved outcomes associated with ANH [7, 13, 14]; the safety of ANH still remains uncertain [15]. We performed this study to assess the relationship between mild volume ANH, perioperative transfusions, and outcomes in Chinese patients undergoing cardiac surgery.

## Methods

### Study population and design

The present study was a single center, retrospective study which was approved by the Ethics Committee of Zhejiang University (Hangzhou, China; No.2016-021), with patient consent waived. A total of 2058 consecutive adult patients underwent cardiac surgeries with CPB from January 1, 2010, to December 31, 2015 and ANH was performed throughout all 5 years. The following exclusion criteria were applied as they were unsuitable for ANH: (1) low weight (weight <50 kg for men and <45 kg for women); (2) ASA (American Standards Association) class V; (3) emergent or redo surgery; (4) previous shock or left ventricular ejection fraction (LVEF) <35%; (5) previous coexistent disease (endocarditis; a history of myocardial infarction in the previous 30 days; chronic obstructive pulmonary disease [COPD]; anemia; chronic kidney disease [CKD]; serum albumin level [ALB] <25 g/L); (6) international normalized ratio (INR) >1.5 or platelet count <100 × 10^3^/mm^3^; (7) acquired or inherited bleeding disorders; (8) cancer disease. Then the study population was devided into ANH group (with ANH) and non-ANH group (without ANH).

Perioperative medical record information including demographic characteristics, patient history (preoperative medications, coexistent disease and other risk factors), operative data, perioperative allogeneic transfusions, and postoperative complication were retrieved from the hospital medical records during the entire hospitalization period regardless of days. An independent investigator reviewed all data.

### ANH techniques and transfusion strategy

General anesthesia and standard monitoring were performed. According to institutional standards, body temperature of patients was maintained at 30 °C during CPB. When the surgery was finished, patients were rewarmed to 37 °C and weaned from CPB. ANH was performed after induction of anesthesia and before heparinization for CPB [7]. The mild volume ANH [8] was performed with 5–8 ml/kg of whole blood withdrawn from an introducer of Fast-Cath catheter (St. Jude Medical, USA) into standard citrate phosphate-dextrose collection bags by gravity. The removed blood volume was simultaneously replaced with an equal volume of hydroxyethyl starch solutions (6%Haes 130/0.4; Fresenius Kabi, Stans, Switzerland). Collected blood was kept at 4 °C temperature refrigerator without shaker.

Transfusion criteria were the same in both groups. The cell saver was routinely performed, the administration of antifibrinolytics was apllied according to the anesthetist’s judgment, and additional blood transfusions were deliberately determined by the surgery team caring of the patients. Blood transfusions were usually carried out when the following criteria were met [2] and other additional decisions were made according to the patient’s clinical condition. Red blood cells (RBC) were transfused when hemoglobin concentrations was <6 g/dL during CPB and <7 g/dL after CPB. When international normalized ratio (INR) >1.4 or activated partial prothrombin time (aPTT) >50 s or R time >10 min in Thromboelastometry test (TEG), prothrombin complex concentrate (PCC) was administered (20–30 IU/kg). If the above mentioned parameters prolongation did not respond to administration of PCC or there occured hypovolemia, Fresh frozen plasma (FFP) was transfused (10–15 mL/kg). Platelets were transfused when platelets count <50 × 10^9^/L and fibrinogen concentrate (Fb) was administered of 25–50 mg/kg when Fb <1.5 g/L. Activated recombinant factor VII (rVIIa) was considered of 90 ug/kg as rescue therapy if the bleeding still existed while INR <1.4, aPTT <50 s, Fb >2 g/L, platelet count >100 × 10^9^/L. When transfusion criteria were met during the surgery, the withdrawn blood was reinfused firstly. If transfusion criteria were not met till the end of surgery, the withdrawn blood was also reinfused before the patients were transferred out of the operation room. The reinfusion of withdrawn blood was performed within 6 h. Blood recovered from the extracorporeal circuit system (pumped blood) was not accounted into the volume of the blood transfusions.

### Perioperative endpoints

The end points occurred during the hospitalization were recorded in patient medical documents. The primary endpoint was any perioperative transfusions. Other endpoints included in-hospital mortality (intraoperation and postoperation death, which were defined as death in the operation room and death after operation up to discharge seperately), morbidity, duration of mechanical ventilation, length of ICU (intensive care unit) stay, length of hospital stay (LOS) and hemostatic drugs adminstration. Hospital morbidities included the following: (1) pulmonary infection was confirmed based on symptoms (fever and expectoration) and laboratory evidence (culture-positive sputum, chest roentgenogram, or computed tomography findings) by a physician reviewing the medical data [16]; (2) prolonged wound healing which was defined as >7 days for wound healing; (3) stroke which was a new ischemic cerebrovascular accident confirmed by radiological evidence with a neurological deficit lasting for more than 24 h and with radiological evidence [17]; (4) new-onset atrial fibrillation (AF) [18]; (5) re-exploration for bleeding during hospitalization; (6) acute kidney injury (AKI), defined by Improving Global Outcomes criteria [19]: increase in serum creatinine (Cr) by ≥26.5 μmol/l within 48 h; or Increase in Cr to ≥1.5 times baseline within the previous 7 days. AKI is staged for severity according to the following criteria: stage 1 (Cr ≥26.5 μmol/L above baseline or 1.5–1.9 times baseline), stage 2 (Cr 2.0–2.9 times baseline), or stage 3 (Cr ≥3 times baseline or postoperative Cr of ≥353.6 μmol/L, or initiation of renal replacement therapy).

## Statistical methods

Descriptive analyses of variables were used to summarize data. The normal distributed variables were expressed as mean ± standard deviation (SD) and compared with student *t*-test. Abnormal continuous variables were expressed as median (interquartile range (IQR)) and evaluated with Mann-Whitney *U*-test.respectively. Chi-square or Fisher’s exact test was used to compare proportions between the two groups. Missing continuous variables of baseline parameters were less than 10% and were replaced by median in Table 1. All reported *p* values were two sided, and values of *p* < 0.05 were considered to be statistically significant. Statistical analysis was performed with SPSS version 18.

**Table 1:** Demographic and Clinical characteristics of the two study groups before and after propensity score matching

	Entire sample			Propensity-matched group		
Preoperative characteristics	ANH		*p*-value	ANH		*p*-value
	Yes (*n* = 358)	No (*n* = 931)		Yes (*n* = 354)	No (*n* = 354)	
Age [median (SD); yr]	50 ± 12	51 ± 13	0.139	50 ± 12	50 ± 13	0.866
Male/female, no.(%)	224/134 (62.6%)	394/537 (42.3%)	<0.001	220/134 (62.1%)	222/132 (62.7%)	0.877
Height [median (SD); cm]	164 ± 7	162 ± 7	<0.001	164 ± 7	164 ± 7	0.761
Weight [mean (SD); kg]	63 ± 10	60 ± 10	<0.001	63 ± 10	62 ± 10	0.580
Estimated blood volume [mean (SD); mL]	4298 ± 779	4045 ± 722	<0.001	4285 ± 765	4258 ± 750	0.636
ASA, no. (%)			0.070			0.230
I	1 (0.3%)	4 (0.4)		1 (0.3%)	3 (0.8%)	
II	79 (22.1%)	206 (22.1%)		79 (22.3%)	77 (21.8%)	
III	248 (69.3%)	678 (72.8%)		244 (68.9%)	256 (72.3%)	
IV	30 (8.4%)	43 (46%)		30 (8.5%)	18 (5.1%)	
NYHA class III/IV, no.(%)	83 (23.2%)	233 (25%)	0.491	81 (22.9%)	75 (21.2%)	0.586
History of smoking, no. (%)	108 (30.2%)	204 (21.9%)	0.002	108 (30.5%)	100 (28.2%)	0.509
Coexistent disease						
AF, no. (%)	81 (22.6%)	222 (23.8%)	0.644	80 (22.6%)	82 (23.2%)	0.858
Hypertension, no. (%)	75 (20.9%)	209 (22.4%)	0.561	74 (20.9%)	79 (22.3%)	0.648
Diabetes, no. (%)	17 (4.7%)	63 (6.8%)	0.179	17 (4.8%)	18 (5.1%)	0.862
HLP, no. (%)	5 (1.4%)	6 (0.6%)	0.191	5 (1.4%)	2 (0.6%)	0.451
Cerebrovascular disease, no. (%)	16 (4.5)	37 (4.0)	0.688	16 (4.5)	16 (4.5)	1.000
Preoperative Medication						
ACEI and ARB, no. (%)	28 (7.8%)	77 (8.3%)	0.792	28 (7.9%)	29 (8.2%)	0.890
β-blockers, no. (%)	19 (5.3%)	66 (7.1%)	0.248	19 (5.4%)	22 (6.2%)	0.629
Calcium Channel Blockers, no. (%)	25 (7.0%)	71 (7.6%)	0.694	24 (6.8%)	19 (5.4%)	0.431
Diuretics, no. (%)	16 (4.5%)	70 (7.5%)	0.049	16 (4.5%)	13 (3.7%)	0.569
Preoperative laboratory examination						
T-ch [median (SD); mmol/L]	4.5 ± 1.0	4.4 ± 1.1	0.178	4.5 ± 1.0	4.4 ± 1.1	0.305
Cr [median (SD); umol/L]	70 ± 15	67 ± 16	<0.001	70 ± 15	70 ± 17	0.976
ALB [median (SD); g/L]	42 ± 4	42 ± 4	0.182	42 ± 4	42 ± 4	0.535
HCT [median (SD); %]	42 ± 5	40 ± 4	<0.001	42 ± 4	42 ± 4	0.918
Propensity score [median (SD)]	0.33 ± 0.12	0.26 ± 0.11	<0.001	0.32 ± 0.12	0.32 ± 0.11	0.840

#: Fisher’s exact test was used; *BMI* body mass index, *ASA* American Society of Anesthesiologists, *NYHA* New York Heart Association, *AF* atrial fibrillation, *HLP* hyperlipidaemia, *CKD* chronic kidney disease, *COPD* chronic obstructive pulmonary disease, *MI* myocardial infarction in 30 days before operation, *ARB* angiotensin receptor blockers, *ACEI* angiotensin converting enzyme inhibitors, *LVEF* left ventricular ejection fraction, *T-ch* serum cholesterol, *Cr* serum creatinine, *ALB* albumin, *HCT* hematocrit

To minimize the effect of selection bias on outcomes, we used propensity score matching for clinical characteristics to reduce distortion by confounding factors. Using propensity score analysis by the method of nearest-neighbor matching, we generated a set of matched cases (ANH) and controls (non-ANH). According to the propensity score matching, 354 pairs of patients were identified for postoperative analysis. A propensity score was generated for each patient from a multivariable logistic regression model on the basis of the covariates using clinical characteristics data (Table 1) from the institutional registry as independent variables, with treatment type (ANH vs. Non-ANH) as a binary dependent variable. We matched patients using a greedy-matching algorithm with a caliper width of 0.1 of the estimated propensity score. A matching ratio of 1:1 was used. We evaluated post match covariate balance by comparing the balance of baseline covariates between patients with ANH and non-ANH before and after matching using absolute standardized differences [20].

## Results

### Baseline parameters

**Fig. 1:**
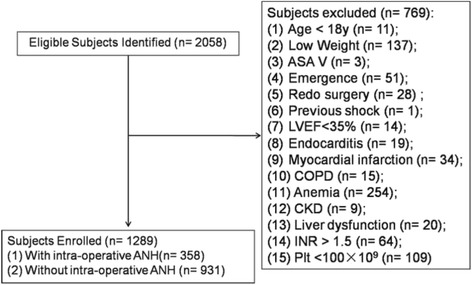
Study population recruitment summary. LVEF = left ventricular ejection fraction; COPD = chronic obstructive pulmonary disease; CKD = chronic kidney disease; INR = international normalized ratio; Plt = platelet count; ANH = acute normovolemic hemodilution

A total of 1289 patients were identified and divided into two groups: patients who received ANH (ANH group, *n* = 358, 27.8%) and those who did not receive ANH (non- ANH group, *n* = 931, 72.2%) during the operative period (Fig. 1). The mean removed blood was 346 mL in the ANH group. The average age of the study population was 51 years, 52.1% were females, 71.8% with ASA class III, 5.7% with ASA class IV, and 24.5% had NYHA class III and IV. A total of 38.7% of the total patients underwent complex cardiac (combined coronary artery bypass graft surgery and valve surgery or multi-valve surgery) or aortic surgery (Aortic dissections, type A and B, thoracic aortic aneurysms or Aortic valve surgery with ascending aortic replacement). The clinical characteristics of the two matched groups (with and without ANH) extracted by propensity analysis were presented in Table 1. According to the standardized difference, covariate balance between the matched pairs was confirmed. An additional data material file shows in detail (see Additional file 1).

### Operative characteristics

As ANH technique was performed, the blood hematocrit was lower in the ANH group (*p* < 0.05). The ANH group had more intraoperative cristalloids and colloids volume (2272 ± 610 vs. 2140 ± 770) mL; *p* = 0.011), but there was no significant difference in blood loss, urine output, and pump blood between the two groups. No significant difference was observed between the two matched groups regarding operative characteristics including surgery type, the number of intra-aortic balloon pump (IABA) utilized, cardiopulmonary bypass time, anesthesia time, surgery time, calcium content, and the blood pH (Table 2).

**Table 2:** Operative Characteristics

Characteristics	ANH		
	Yes (*n* = 354)	No (*n* = 354)	*p*-value
ANH [mean (SD); mL]	342 ± 138		
Operative Characteristics			
Intra-aortic balloon pump, no. (%)	1 (0.3%)	1 (0.3%)	1.000
Duration of anesthesia [mean (SD); min]	297 ± 98	305 ± 110	0.325
Duration of surgery [mean (SD); min]	252 ± 89	260 ± 110	0.244
CPB time [mean (SD); min]	125 ± 59	129 ± 63	0.399
Type of surgery, no. (%)			0.168
CABG	23 (6.5%)	30 (8.5%)	
Valve surgery	137 (38.7%)	121 (34.2%)	
Complex cardiac surgery	116 (32.8%)	126 (35.6%)	
Aortic surgery	24 (6.8%)	13 (3.7%)	
Others surgery	54 (15.3%)	64 (18.1%)	
Input and Output			
Blood loss [mean (SD); mL]	684 ± 355	678 ± 514	0.855
Urine output [mean (SD); mL]	788 ± 465	730 ± 419	0.084
Pump blood [mean (SD); mL]	512 ± 129	508 ± 139	0.708
Cell salvage [median (SD); mL]	375 ± 267	369 ± 343	0.797
Intraoperative cristalloids and colloids [mean (SD); mL]	2272 ± 610	2140 ± 770	0.011
Hemostatic drugs Characteristics			
Tranexamic Acid, no. (%)	121 (34.2%)	113 (31.9%)	0.523
Prothrombin Complex Concentrate, no. (%)	6 (1.7%)	7 (20%)	0.780
Fibrinogen concentrate, no. (%)	6 (1.7%)	10 (2.8%)	0.312
Recombinant activated factor VII, no. (%)	1 (0.3%)	3 (0.8%)	0.624
Arterial blood gas analysis			
Calcium content Pre-CPB [mean (SD); mmol/L]	1.08 ± 0.10	1.08 ± 0.09	0.871
Blood pH Pre-CPB [mean (SD)]	7.40 ± 0.06	7.39 ± 0.05	0.636
Hct Pre-CPB [mean (SD);%]	38 ± 5	40 ± 5	<0.001
Calcium content End ± CPB [mean (SD); mmol/L]	0.90 ± 0.13	0.87 ± 0.13	0.086
Blood pH End-CPB [mean (SD)]	7.48 ± 0.08	7.50 ± 0.09	0.055
Hct End-CPB [mean (SD);%]	25 ± 4	27 ± 5	0.003
Calcium content Post ± CPB [mean (SD); mmol/L]	1.03 ± 0.17	1.02 ± 0.15	0.628
Blood pH Post-CPB [mean (SD)]	7.37 ± 0.07	7.38 ± 0.08	0.168
Hct Post ± CPB [mean (SD);%]	29 ± 4	30 ± 5	0.021

#: Fisher’s exact test was used; *CPB* cardiopulmonary bypass, *CABG* coronary artery bypass grafting, *Valve surgery* aortic, mitral and tricuspid valve surgery without ascending aortic replacement, *Complex cardiac surgery* combined coronary artery bypass graft surgery and valve surgery or multi-valve surgery, *Aortic surgery* aortic dissections, type A and B, thoracic aortic aneurysms) or Aortic valve surgery with ascending aortic replacement; Others surgery type including atrial septal defect, interventricular septal defect, atrial myxoma, Aneurysm Sinus Valsalva, coronary artery pulmonary artery fistula, patent foramen ovale/atrial septal aneurysm surgery, and surgery for cardiac tumors, *Pumped blood* blood recovered from the extracorporeal circuit system, *CPB* cardiopulmonary bypass, *Hb* hemoglobin, *Hct* hematocrit, *Pre-CPB* before CPB and after performing ANH, *End-CPB* at the end of CPB, *Post-CPB* 30 min after CPB

### Perioperative allogeneic transfusions

Of the total 1289 patients, 500 patients (38.8%) received perioperative RBC transfusions, 10% (129/1289) of patients received platelet, 56.4% (727/1289) of patients received FFP transfusions. Compared to the non-ANH group, the intraoperative RBC transfusions rate (8.5% vs. 14.4%; *p* = 0.013) and number of RBC units (*p* = 0.019) decreased significantly in the ANH group. However, there was no significant difference regarding intraoperative hemostatic drugs, FFP and platelet concentrate transfusions, as well as postoperative and total perioperative allogeneic transfusions (Table 3).

**Table 3:** Perioperative allogeneic transfusions

Allogeneic transfusions	Propensity-matched		
	ANH	Non-ANH	*p*-value
	(*n* = 354)	(*n* = 354)	
Intra-operative transfusion			
RBC transfusion, no. (%)	30 (8.5%)	51 (14.4%)	0.013
Total RBCs transfusion (U)	164	289	0.019
FFP transfusion, no. (%)	96 (27.1%)	109 (30.8%)	0.281
Total FFP transfusion (ml)	63770	78660	0.225
Platelet transfusion, no. (%)	25 (7.1%)	18 (5.1%)	0.271
Total Platelet transfusion (U)	261	205.7	0.432
Postoperative RBCs transfusion			
Postoperative in 24 h, no. (%)	72 (20.4%)	63 (17.8%)	0.389
Postoperative in 24 h [median (IQR); U]	0 (0 to 0)	0 (0 to 0)	0.407
>24 h after surgical start, no. (%)	24 (6.8%)	25 (7.1%)	0.882
>24 h after surgical start [median (IQR); U]	0 (0 to 0)	0 (0 to 0)	0.881
Postoperative FFP transfusion			
Postoperative in 24 h, no. (%)	90 (25.5%)	93 (26.3%)	0.797
Postoperative in 24 h [median (IQR); ml]	0 (0 to 230)	0 (0 to 200)	0.914
Postoperative after 24 h, no. (%)	7 (2.0%)	7 (2.0%)	1.000
>24 h after surgical start [median (IQR); ml]	0 (0 to 0)	0 (0 to 0)	0.996
Postoperative Platelet transfusion			
Postoperative in 24 h, no. (%)	10 (2.8%)	9 (2.5%)	0.816
Postoperative in 24 h [median (IQR); U]	0 (0 to 0)	0 (0 to 0)	0.802
Postoperative after 24 h, no. (%)	1 (0.3%)	2 (0.6%)	0.624#
>24 h after surgical start [median (IQR); U]	0 (0 to 0)	0 (0 to 0)	0.561
Total Perioperative Allogeneic transfusions			
RBC transfusion, no. (%)	98 (27.7%)	96 (27.1%)	0.866
RBCs transfusion (U)	0 (0 to 2)	0 (0 to 2)	0.300
FFP transfusion, no. (%)	153 (43.2%)	157 (44.4%)	0.762
FFP transfusion (ml)	0 (0 to 730)	0 (0 to 630)	0.876
Platelet transfusion, no. (%)	35 (9.9%)	28 (7.9%)	0.355
Platelet transfusion (U)	0 (0 to 0)	0 (0 to 0)	0.380
Postoperative HCT [median (SD); %]	32 ± 5	32 ± 4	0.464

*RBCs* red blood cells, *FFP* fresh frozen plasma, *HCT* hematocrit, *ANH* acute normovolemic hemodilution

### Postoperative outcomes after propensity matching

Eighteen of the total 1289 patients (1.4%) died during hospitalization, of which died in the operating room were four. Patients who died in the operating room after propensity matching were excluded from the postoperative outcomes analysis (*n* = 2). Patients who had preexisting renal dysfunction (serum creatinine level >124 μmol/L for women and >141 μmol/L for men or requiring renal replacement therapy) were excluded from the AKI analysis (*n* = 52) and patients with a preexisting history of AF were excluded from the AF analysis after propensity score matching (*n* = 161).

None of the patients experienced pulmonary embolism. Approximately 8.9% (115/1289) of patients developed postoperative pulmonary infection during hospitalization. The rate of pulmonary infection (6.8 vs. 11.3%; *p* = 0.036) was significantly declined in the ANH group as compared to that in the non-ANH group. No differences were found in the incidence of mortality, prolonged wound healing, stroke, AF, reoperation for postoperative bleeding and AKI between the two groups. There was also no difference in postoperative ventilation time, length of ICU and hospital stay (Table 4).

**Table 4:** Postoperative outcomes (*n* = 354)

Outcomes	Propensity-matched		
	ANH	Non-ANH	*p* valve
Pulmonary infection			
no	329 (93.2%)	313 (88.7%)	
yes	24 (6.8%)	40 (11.3%)	0.036
Death			
no	350 (99.2%)	351 (99.4%)	
yes	3 (0.8%)	2 (0.6%)	1.000#
Prolonged wound healing			
no	332 (94.1%)	341 (96.6%)	
yes	21 (5.9%)	12 (3.4%)	0.109
Stroke			
no	352 (99.7%)	350 (99.2%)	
yes	1 (0.3%)	3 (0.8%)	0.624#
AF			
no	237 (86.8%)	233 (85.7%)	
yes	36 (13.2%)	39 (14.3%)	0.696
Resternotomy for postoperative bleeding			
no	349 (98.9%)	350 (99.2%)	
yes	4 (1.1%)	3 (0.8%)	1.000#
AKI			
no	245 (74.0%)	241 (74.6%)	
yes	86 (26.0%)	82 (25.4%)	0.862
Stage1	63 (73.3%)	59 (71.9%)	
Stage2	15 (17.4%)	18 (22.0%)	
Stage3	8 (9.3%)	5 (6.1%)	
Ventilation [mean (SD); hours]	17 ± 21	19 ± 29	0.239
ICU [mean (SD); days]	4.4 ± 2.4	4.2 ± 3.1	0.254
LOS [mean (SD); days]	12 ± 9	12 ± 10	0.708

*AF* atrial fibrillation, *AKI* acute kidney injury, *ANH* acute normovolemic hemodilution, *ICU* intensive care unit, *LOS* length of hospital stay

## Discussion

In our retrospective analysis of patients undergoing cardiac surgery with CPB, we found that mild volume ANH was associated with decreased intraoperative RBC transfusions rate and number of RBC units after data adjustment for preoperative risk factors. However, there was no significant difference regarding postoperative and total perioperative allogeneic transfusions. Our results further supported previous findings that the use of ANH could reduce intraoperative RBC transfusions in patients undergoing cardiac surgery [6, 7, 21], even though blood loss was similar between the ANH and non-ANH groups in our study. Some meta-analysis also supported that ANH is effective in minimizing blood transfusion in patients undergoing cardiac surgery [15, 22].

However, the utility of mild volume ANH in reducing allogeneic blood transfusions in cardiac surgery is still controversial. Several studies have proved that mild volume ANH was not effective in reducing the number of allogeneic erythrocytes units [8, 23], but others have proven otherwise [6]. Our results support the positive finding that mild volume ANH could reduce intraoperative blood transfusions. The variations in blood saving strategy and surgery type may be the major reasons for our finding being different from other studies. Although Valter Casati et al.’s study did not find positive efficacy of low-volume ANH [8], we noticed that there was a decreasing trend of intraoperative RBC transfusions in the ANH group compared to non-ANH group (4.9% vs. 7%). Another potential reason was that their case mix was easier (over 50% were single valve surgeries), which leads to less blood loss and transfusions. However, more than 38.7% of the surgeries in our cohort were complex cardiac and aortic surgeries, resulting in that more patients required intraoperative RBC transfusions in both the ANH and non-ANH groups (8.5 and 14.4%). Another point to be noted is that the cell saver technique was routinely applied in our study as a previous study reported that combination of cell saver with ANH produced better blood saving effect than ANH alone [24]. Another previous study of Alireza Mahoori et al. on exclusively elective coronary artery bypass graft (CABG) surgery patients [6] found that mild volume ANH could decrease requirements of total perioperative RBC transfusions. We failed to observe the similar positive effect of mild volume ANH on total perioperative RBC transfusions, this variation may mainly due to the different volumes of blood removed (342 vs. 490 mL) and transfusion triggered criteria between our study and Alireza Mahoori^’^s study (hemoglobin concentration <6–7 g/dL vs. <10 g/dL). The less strict transfusion triggered criteria in Alireza Mahoori^’^s study may lead to more transfusions rate compared to our cohort (57% vs. 27%).

Although ANH has been proved to reduce postoperative allogenic blood transfusions during cardiac surgery [7], our study failed to show these positive findings. A comprehensive blood saving strategy including antifibrinolytic agent [25] and TEG [26] has been applied and was demonstrated to reduce transfusion requirements. Thus the evidence of ANH may be less effective. The mild volume of ANH may also contribute to the less beneficial blood conservation effect, as Joshua Goldberg found that the reduction in allogeneic transfusions is more significant with greater volume ANH (≥800 mL) in cardiac surgery [7].

There is not much evidence regarding clinical outcome benefits of mild ANH in patients undergoing cardiac surgery. A recent article found that ANH was related to improved outcomes including renal failure, 30-day mortality, and length of hospital stay [7], and moderate ANH provided further cardioprotective effects during cardiac surgery [13, 14]. However, some other authors mentioned that greater hemodilution might be associated with adverse complications, especially in patients with preexisting coronary disease [27]. In another study of patients undergoing pancreaticoduodenectomy, ANH did not seem to decrease blood transfusions, but lead to more pancreatic anastomotic complications for greater intraoperative fluid administration [28]. Some authors criticize ANH for the possibly of causing perioperative adverse outcomes including damaged pulmonary function for increased lung fluid volume [8]. Furthermore, a recent study even found that after moderate ANH, patients undergoing total hip arthroplasty showed a hypocoagulable state [29].

Although we did not observe the positive effect of ANH regarding mortality, stroke, AF, AKI, and resternotomy for postoperative bleeding, our findings suggest that mild volume ANH might be associated with lower incidence of pulmonary infection. Some possible mechanisms may contribute to this protective effect. First, the indirect effect of reducing the rate of RBC transfusions may improve outcomes. RBC transfusions could increase postoperative pneumonia in patients undergoing CABG surgery [17]. Second, ANH blood was associated with a smaller concentration of inflammatory mediators such as interleukin-10 and neutrophil elastase than predonated autologous blood [30]. Third, due to the hemodilution-induced blood viscosity, such protection might be associated with an improved oxygen supply-consumption balance [14]. More evidence is needed to confirm the positive effect of mild volume ANH on cardiac surgery outcomes, including pulmonary complication.

### Limitations

There were some limitations in this study. Firstly, due to clear and strict indications, ANH [2] could not be performed widely for all paitents. Secondly, although the strict exclusion criteria and propensity score adjustment were assumed to minimize biases, the potential bias factors may not be completely eliminated in a retrospective study. Thirdly, although no significant difference was observed between the two matched groups regarding intraoperative tranexamic acid adminstration, the administration of tranexamic acid was not absolutely unified in this study. Finally, population characteristics and the blood saving strategy were different for each institution; hence, caution should be used when interpreting our findings.

## Conclusions

In conclusion, in our large retrospective study, mild volume ANH was associated with lower intraoperative RBC transfusions and postoperative pulmonary infection in Chinese patients undergoing cardiac surgery. However, there was no significant difference regarding postoperative and total perioperative allogeneic transfusions. Further prospective and randomized studies are needed to confirm the effects of mild volume ANH on perioperative outcomes during cardiac surgery.

## Additional file

Additional file 1: Study Data Information. Description of data: Details of 1289 adult patients underwent cardiac surgeries with CPB from January 1, 2010, to December 31, 2015 after excluding those who were unsuitable for performing ANH (XLS 1175 kb)

## Abbreviations

AF: Atrial fibrillation
AKI: Acute kidney injury
ALB: Albumin
ANH: Acute normovolemic hemodilution
aPTT: Activated partial prothrombin time
ASA: American standards association
CKD: Chronic kidney disease
COPD: Chronic obstructive pulmonary disease
CPB: Cardiopulmonary bypass
Cr: Creatinine
Fb: Fibrinogen concentrate
FFP: Fresh frozen plasma
IABA: Intra-aortic balloon pump
ICU: Intensive care unit
INR: International normalized ratio
LOS: Length of hospital stay
LVEF: Left ventricular ejection fraction
RBC: Red blood cells
rVIIa: Recombinant factor VII
TEE: Transesophageal echocardiography
TEG: Thrombelastography
